# Clinical value of serum sTREM-1 and HBP levels in combination with traditional inflammatory markers in diagnosing hospital-acquired pneumonia in elderly

**DOI:** 10.1186/s12879-022-07758-9

**Published:** 2022-10-04

**Authors:** Zhang Wang, Binbin Chang, Yong Zhang, Jieyu Chen, Fang Xie, Ying Xiang, Tingting Liu, Ying Li

**Affiliations:** Department of Geriatrics, The General Hospital of Western Theater Command, No. 270 Rongdu Road, 610083 Chengdu, Sichuan China

**Keywords:** Biomarker, sTREM-1, HBP, Hospital-acquired pneumonia, Elderly

## Abstract

**Background:**

The clinical presentation of hospital-acquired pneumonia (HAP) in older patients is often complex and non-specific, posing a diagnostic challenge. This study evaluates the value of serum soluble triggering receptor expressed on myeloid cells-1 (sTREM-1) and heparin-binding protein (HBP) in combination with traditional inflammatory markers procalcitonin (PCT) and C-reactive protein (CRP) in diagnosing HAP in older patients.

**Methods:**

Thirty-eight elderly male patients with HAP (≥ 80 years old) and 46 age-matched controls, who were hospitalized for other reasons than HAP, were enrolled. The serum sTREM-1, HBP, PCT and CRP levels were measured by ELISA on the first day after enrollment. In addition, routine blood test, blood gas, sputum analysis, clinical pulmonary infection score (CPIS) assessment, and chest X-ray were performed, and the correlations with HAP were analyzed.

**Results:**

The serum sTREM-1 (*n* = 38, 170.75 ± 158.33 pg/ml), HBP (2.08 ± 0.50), PCT (9.44 ± 17.73) and CRP (79.63 ± 71.37) were all significantly higher in the HAP group, when compared to the control group (*P* < 0.05). Furthermore, the values were positively correlated with the CPIS. The ROC curve analysis revealed that the AUC for sTREM-1 (0.667) and HBP (0.711) were lower, when compared to that for PCT (AUC = 0.839) and CRP (AUC = 0.840). The combination of PCT and CRP with sTREM-1 (AUC = 0.927) or HBP (AUC = 0.930) had the highest AUC values.

**Conclusion:**

Serum sTREM-1, HBP, PCT and CRP can all be used as diagnostic markers for HAP in the elderly. The combination of traditional inflammatory markers PCT and CRP with novel inflammatory marker sTREM-1 or HBP further improves the diagnostic performance.

## Background

Pneumonia is one of the most common infections in the elderly. The mortality and morbidity of pneumonia are higher in older patients, when compared to younger patients, due to the deterioration of the immune system with age [1]. Hospital-acquired pneumonia (HAP) is defined as pneumonia that develops at ≥48 h after admission to the hospital. The patient should not be within the incubation phase of the infection, and should not be intubated or ventilated at the time of the infection. Furthermore, the longer the hospital stay, the greater the risk of contracting difficult-to-treat antibiotic-resistant bacterial strains. HAP is associated with extended length of hospital stay and higher mortality rates, especially in older patients [2].

The early diagnosis of HAP is crucial for improving treatment outcomes and survival. At present, there are no universally accepted gold standard diagnostic criteria for HAP. Hence, the clinical diagnosis is based on the comprehensive evaluation of the patient’s condition, including the symptoms, signs on the radiological examination, sputum analysis, blood biomarkers, etc. Due to the insidious onset and atypical clinical manifestations of HAP, especially in elder patients, its early and correct diagnosis is often difficult. Various factors can interfere with the doctor’s judgment, such as non-infectious fever, atypical changes in lung radiology, and co-morbidity with cardiovascular or other lung diseases that can present with symptoms that are similar to pneumonia. Microbiological analyses, such as sputum culture, requires time, and the risk of false-negative results is high [3]. Therefore, there is an urgent need for novel biomarkers that can improve the existing diagnostic process of HAP.

Triggering receptor expressed on myeloid cell-1 (TREM-1) belongs to the immunoglobulin superfamily, and is expressed in neutrophils, monocytes and macrophages. There are two forms of TREM-1: cell membrane-bound or soluble. Studies have revealed that soluble TREM-1 (sTREM-1) is released into the blood or body fluids during acute inflammatory reactions caused by infections. Therefore, it has been suggested that sTREM-1 can function as a biomarker for the identification of infections and sepsis [4, 5]. For patients with ventilator-associated pneumonia (VAP), sTREM-1 is significantly elevated in both the bronchoalveolar lavage fluid (BALF) and exhaled breath condensate (EBC) [6]. It was also reported that serum sTREM-1 is significantly elevated in patients with community-acquired pneumonia (CAP) and sepsis, and that the level of sTREM-1 is correlated with disease severity [7].

Heparin-binding protein (HBP) is another novel inflammatory factor released from neutrophils. HBP is pre-produced and stored in secretory vesicles in neutrophils. When there is an infection or inflammatory process, neutrophils are activated, and HBP is rapidly released from the storage vesicles. It was reported that HBP can directly kill both Gram-positive and Gram-negative bacteria, and further enhance bacterial clearance by attracting macrophages, lymphocytes, and even more neutrophils to the site of infection [8]. Hence, HBP was suggested as a novel and valuable biomarker for sepsis and interstitial lung disease complicated by infection [9, 10].

In the present study, the serum sTREM-1 and HBP levels were quantified in elderly patients with HAP. The value of sTREM-1 and HBP, either alone or in combination with the patient’s clinical pulmonary infection score (CPIS) [11] and traditional inflammatory indicators (PCT and CRP) for the clinical diagnosis of HAP in the elderly, were explored.

## Methods

### Participants

Thirty-eight elderly (≥ 80 years old) male HAP patients, who were hospitalized between June 2018 and December 2021, were enrolled for the present study. Patients with the following conditions were excluded: CAP, bronchiectasis, acute tuberculosis, lung cysts, other respiratory diseases, or infections in other organs.

The control group consisted of age-matched non-HAP patients, who were hospitalized in the same hospital during the same period, but had other diagnoses.

The present study was performed in accordance with the principles of the Declaration of Helsinki, and approved by the Ethics Committee of The General Hospital of Western Theater Command PLA (2021EC1-7). All methods were carried out in accordance with relevant guidelines and regulations. An informed consent was obtained for each enrolled patient. If the patient was unable to provide an informed consent, the consent was obtained from the legal guardian.

### Diagnosis

HAP was diagnosed according to the 2005 guidelines for “Adult Hospital-Acquired Pneumonia, Ventilator-Associated Pneumonia and Medical Care” from the Infectious Diseases Society of America (IDSA) [12]. A simplified CPIS [13], which included temperature, blood leukocyte count, tracheal secretions, oxygenation, and the characteristics of the pulmonary infiltrate on chest X-ray, was employed. All enrolled patients had a CPIS of > 6.

### Blood tests

Peripheral venous blood (5 ml) was collected from all participants on the 1st day after enrollment. For HAP patients, the first day of enrollment was the day the diagnosis of HAP was made. Within 60 min of collection, the samples were centrifuged (4,000 r/min) for five minutes, and the separated serum was stored in a -70 °C freezer for the subsequent analysis.

The serum sTREM-1 and HBP concentrations were quantified by double-antibody sandwich enzyme-linked immunosorbent assay (ELISA). The ELISA kits were purchased from Elabscience Biotechnology Co. Ltd. Routine measurements for PCT, CRP, prealbumin (PA), and white blood cell (WBC) levels were carried out by the laboratory of the hospital.

### The clinical pulmonary infection score

The clinical data of the HAP patients were collected and independently evaluated by two senior physicians to obtain the CPIS. The physicians had no knowledge of the blood test results during the scoring. The average score given by the two physicians was recorded.

### Statistical analysis

All data were analyzed using the SPSS 18.0 statistical software. Normally distributed variables were expressed as mean ± standard deviation (x ± SD). *T*-test was used to compare continuous variables between two groups, and chi-squared test was used to compare the count data between two groups. Receiver operating characteristic (ROC) curves were drawn to calculate the sensitivity, specificity, and area under the curve (AUC). DeLong’s test was performed to compare the results between single marker and the combination of multiple markers. The correlations between variables and outcomes were calculated using Spearman’s correlation analysis. *P* < 0.05 was considered statistically significant.

## Results

### Demographics and clinical characteristics

The basic demographics and clinical characteristics, such as age, comorbidities and CPIS values, were collected for the 38 elderly male HAP patients and 46 age-matched controls. In addition, the 28-day survival was recorded. The detailed clinical data is presented in Table 1.

**Table 1:** Demographics and clinical characteristics

Characteristics	HAP group (*n* = 38)	Control group (*n* = 46)	*P*-value
Age	89.01±5.83	88.03±4.20	0.379
CPIS score	8.45±1.69	2.50±1.44	< 0.001
Comorbidities, *n* (%)			
COPD	11 (29)	12 (26)	n.s.
Diabetes mellitus type 2	10 (26)	10 (22)	n.s.
Heart failure	8 (21)	9 (20)	n.s.
Hypertension	13 (34)	17 (37)	n.s.
Chronic renal disease	6 (16)	5 (11)	n.s.
Solid tumor malignancy	4 (11)	5 (11)	n.s.
28-day mortality, *n* (%)	13.0 (34.2)	3.0 (6.5)	0.018

n.s.: not significant

The average age of patients was high in both groups: 89.01 years old in the HAP group and 88.03 years old in the control group. All enrolled patients were above 80 years old. Comorbidities were common among the enrolled patients, and there were no significant differences in comorbidities between the two groups. Compared to the control group, patients in the HAP group had significantly higher CPIS results, and had over five times higher 28-day mortality rates (34.2% vs. 6.5%).

### Level of inflammatory markers

Compared to the control group, the HAP group had statistically significantly higher levels of sTREM-1, HBP, PCT, CRP and WBC. However, the PA level was significantly lower in the HAP group, when compared to the control group, since the PA level is low when the inflammation level is high. The greatest difference was observed in the PCT values, in which the HAP group had an 18.88-fold higher value, when compared to the control group. The detailed results are presented in Table 2.

**Table 2:** Levels of serum inflammatory markers

	HAP group (*n* = 38)	Control group (*n* = 46)	Fold change	*P*-value
sTREM-1	170.75±158.33	86.56±18.75	1.97	0.002
HBP	2.08±0.50	1.56±0.76	1.33	< 0.001
PCT	9.44±17.73	0.50±1.33	18.88	0.001
CRP	79.63±71.37	19.15±32.85	4.15	< 0.001
PA	166.81±59.17	196.00±58.08	0.88	0.027
WBC	10.51±5.58	6.76±2.46	1.55	< 0.001

### ROC curve analysis of single inflammatory markers

**Fig. 1:**
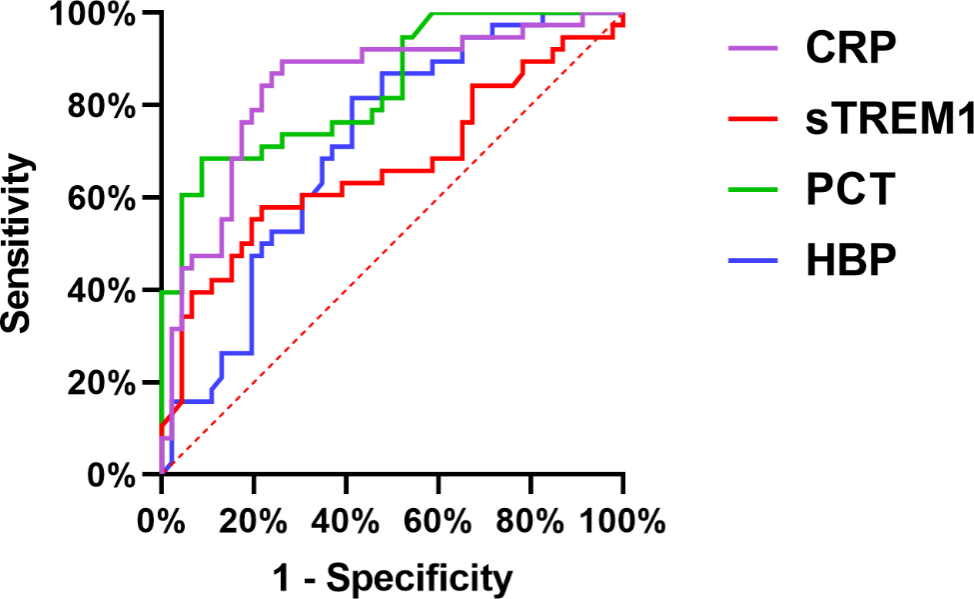
ROC curve analysis of single inflammatory markers

Figure 1 presents the ROC curve analysis for serum sTREM-1, HBP, PCT and CRP. The closer the AUC was to 1.0, the better the diagnostic performance: when the AUC was within 0.5–0.7, the diagnostic accuracy was considered to be low; when the AUC was within 0.7–0.9, the diagnostic accuracy was considered acceptable; when the AUC was > 0.9, the diagnostic accuracy was considered to be high. As shown in Table 3, novel inflammatory markers sTREM-1 and HBP did not have a high diagnostic accuracy, while traditional inflammatory markers CRP and PCT had an acceptable diagnostic accuracy.

**Table 3:** AUC for serum sTREM-1, HBP, PCT, CRP and their combinations

Variable	Area	Standard error^a^	Sig (*P*-value)^b^	Upper 95% CI	Lower 95% CI
PCT	0.839	0.043	0.000	0.754	0.923
CRP	0.840	0.045	0.000	0.752	0.929
sTREM-1	0.667	0.062	0.009	0.546	0.788
HBP	0.711	0.056	0.001	0.601	0.821
PCT + CRP	0.911	0.034	0.000	0.844	0.977
HBP + sTREM-1	0.771	0.051	0.000	0.672	0.871
PCT + CRP + sTREM-1	0.927	0.031	0.000	0.866	0.988
PCT + CRP + HBP	0.930	0.028	0.000	0.875	0.985

a: Assumed as non-parametric; b: Null hypothesis: solid area = 0.5

### ROC curve analysis of the combination of inflammatory markers

**Fig. 2:**
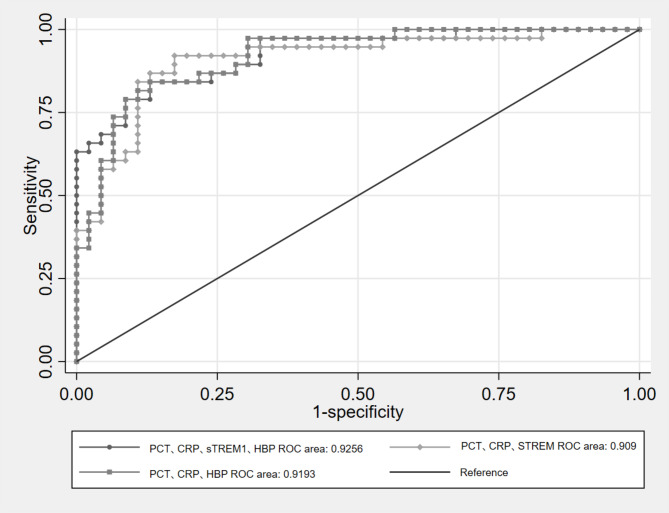
ROC curve analysis of the combination of inflammatory markers

The combination of inflammatory markers led to a significantly higher AUC, when compared to individual inflammatory markers. The PCT + CRP combination caused the AUC to raise above 0.9. The further addition of sTREM-1 or HBP to the PCT + CRP combination caused the AUC to rise even higher (at 0.927 and 0.930, respectively). As shown in the tables, the combined use of PCT, CRP, sTREM and HBP had the highest value (AUC = 0.9256), but this was not significantly better, when compared to the use of the combination of merely three markers (*P* = 0.33 and 0.62). However, the HBP + sTREM-1 combination merely caused the AUC to reach 0.771, which was not significantly higher, when compared to HBP alone. The detailed data is presented in Tables 3 and 4; Fig. 2.

**Table 4:** DeLong’s test for comparison of AUCs for the combinations

Marker combination	ROC area	Standard error^a^	*X* ^*2*^	df	Pr > *X*^*2*^
HBP + sTREM-1 + PCT + CRP (standard)	0.9256	0.0277			
PCT + CRP + sTREM-1	0.9090	0.0335	0.9250	1	0.3362
PCT + CRP + HBP	0.9193	0.0289	0.2501	1	0.6170

a: Assumed as non-parametric

## Spearman’s correlation analysis between sTREM/HBP and outcomes

**Fig. 3:**
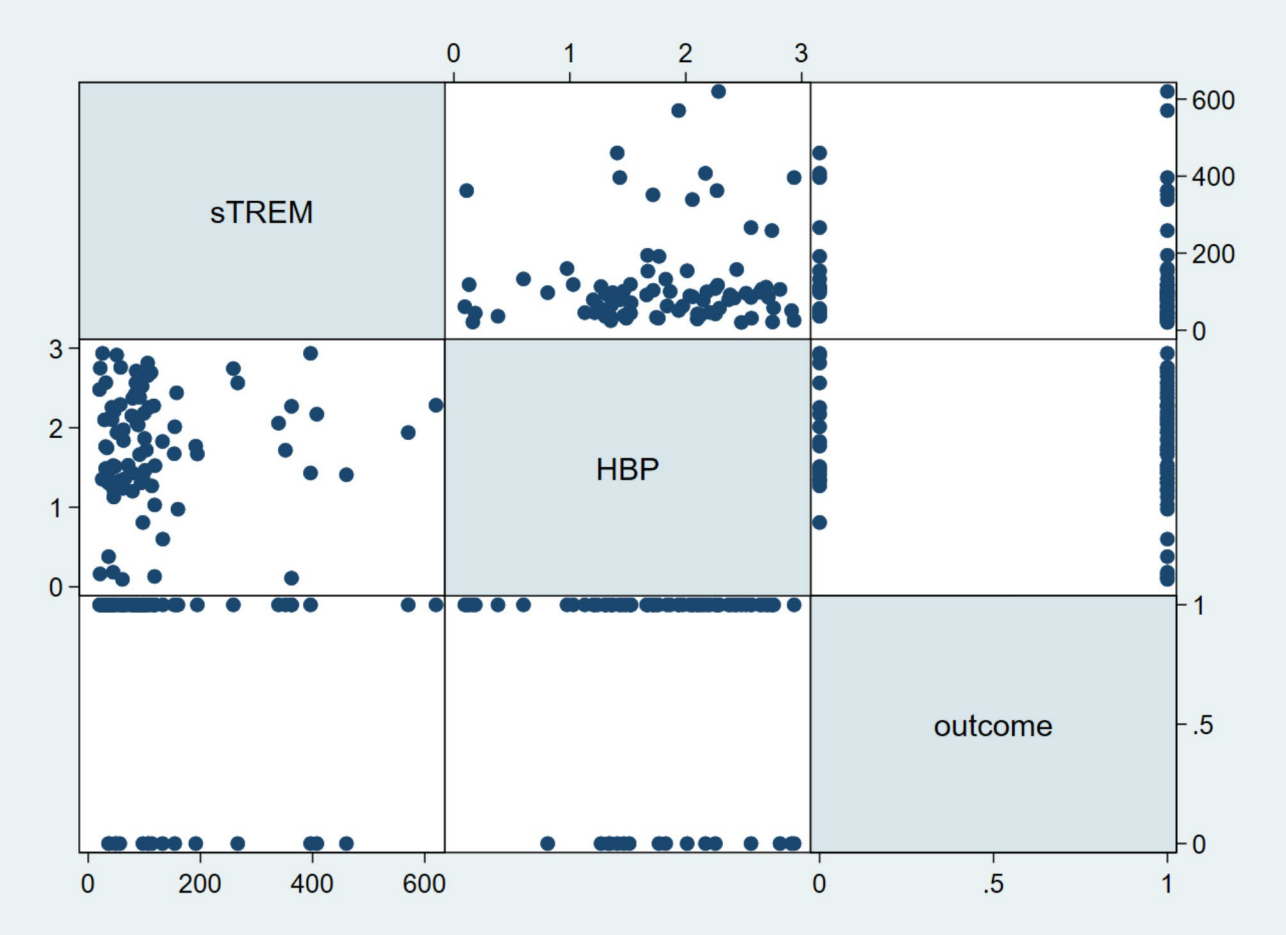
Correlation analysis between sTREM/HBP and outcomes

The correlation analysis did not reveal a significant correlation between sTREM/HBP and outcomes for patients with HAP (*P* > 0.05, Fig. 3).

## Discussion

HAP, including VAP, poses a diagnostic challenge to clinicians worldwide. Its correct and timely diagnosis remains especially difficult in the elderly due to atypical clinical manifestations and interference from other clinical factors not related to the infection itself, such as comorbidities. Furthermore, the present diagnosis is largely based on the evaluation of the patient’s clinical condition, such as fever, leukocytosis, cough and sputum production, in combination with radiological characteristics. Inflammatory biomarkers have exhibited increasing value as an aid in the diagnosis of HAP. In 2018, the Chinese guidelines for HAP/VAP recommended the use of PCT and CRP to increase the diagnostic accuracy of HAP. CRP is clearly elevated during infections, but has low specificity. PCT has higher specificity for bacterial infections, when compared to CRP, but its diagnostic accuracy can be affected by prior exposure to antibiotics [14]. Various studies are presently focusing on the use of novel infection-related inflammatory biomarkers in the diagnosis of HAP.

The present study revealed that novel inflammatory markers sTREM-1 and HBP can both aid in the diagnosis of HAP in the elderly with acceptable accuracy. These results are in line with the results reported by a previous study conducted by Sathe et al. [15] on critically ill patients in the intensive care unit. This study revealed that for patients > 50 years old, the increase in sTREM-1 was more prominent when the patient was older, suggesting that sTREM-1 might be an especially useful biomarker in older patients, when compared to younger patients. Terraneo et al. [16] reported that patients with pulmonary infections have higher levels of sTREM-1 in both plasma and BALF, when compared to healthy controls. Radsa et al. [17] reported that the level of sTREM-1 in plasma is positively correlated to the degree of inflammation in the lungs, suggesting that sTREM-1 might participate in the inflammatory cascade, and cause damage to lung tissues. Furthermore, the expression and activities of receptors and cytokines are significantly increased in elderly patients with CAP. Porfyridis et al. [18] reported that the average level of sTREM-1 in serum was 102.09 pg/ml for patients with CAP, and merely 15.1 pg/ml for patients with non-bacterial pulmonary disease. Using 19.53 pg/ml for sTREM-1 as the cut-off point, the diagnostic sensitivity was 82.5% and the diagnostic specificity was 63%. This shows that sTREM-1 is an important serum marker for bacterial lung infections. Vimal Grover et al. [19] investigated the levels of sTREM-1 in the blood and BALF of VAP patients, and reported that these levels were significantly higher, when compared to control subjects (*P* < 0.001). Hence, the use of multiple biomarkers, including sTREM-1, can accurately help to distinguish VAP patients from those not affected by any pulmonary infection [20].

HBP has been previously reported to have a clear diagnostic value in children with acute bacterial infection [21]. The present study revealed that HBP can also be useful for older patients with HAP. For patients with interstitial lung disease (ILD), HBP has been shown to be useful in detecting acute infections. Patients with acute infection have significantly higher levels of HBP, when compared to patients with a stable disease. Furthermore, HBP has been shown to have higher accuracy, when compared to CRP and PCT, in detecting pulmonary infections in ILD [10] For patients with COVID-19 infection, HBP is significantly elevated, but this level is negatively correlated with oxygen saturation, creatinine and lactate levels. Moreover, it was reported that HBP is an independent risk factor for death within 28 days [22].

However, compared to traditional inflammatory markers, such as PCT and CRP, the present study revealed that serum sTREM-1 and HBP alone do not exhibit higher diagnostic accuracy for HAP in elderly patients, and that the sensitivity and specificity were not better, when compared to PCT and CRP.

A previous study revealed that the combination of novel inflammatory marker HBP with traditional inflammatory markers PCT and CRP can lead to significantly higher diagnostic accuracy, when compared to any of these markers alone [14]. Furthermore, the present study revealed that the combination of inflammatory markers exhibited a significantly improved diagnostic accuracy. That is, the PCT + CRP + sTREM-1 and PCT + CRP + HBP combinations had the highest AUC values, thereby having the highest diagnostic accuracy.

At present, although there are increasing reports on the diagnostic value of sTREM-1 and HBP in infection- and inflammation-related diseases, the reported results were not consistent [23]. The difference in study population, study design, and experimental conditions may have affected the results. Unfortunately, merely male patients were included for the present study, since the study was carried out in a hospital for veterans, and the percentage of female patients at the hospital was merely approximately 5%. Therefore, more research, preferably multicenter studies, with both male and female patients, are needed to further explore the clinical application value of sTREM-1 and HBP.

## Conclusion

Serum sTREM-1 and HBP are novel inflammatory markers that are significantly elevated in elderly patients with HAP. The combination of traditional inflammatory markers PCT and CRP with novel inflammatory marker sTREM-1 or HBP can significantly improve the diagnostic performance.

## Data Availability

The datasets generated and/or analyzed for the present study are not publicly available due to limitations on the ethics approval, which involves patient data and anonymity. However, these may be made available from the corresponding author on reasonable request.

## List of abbreviations

HAP: hospital-acquired pneumonia;
sTREM-1: serum soluble triggering receptor expressed on myeloid cells-1;
HBP: heparin-binding protein;
PCT: procalcitonin;
CRP: C-reactive protein;
CPIS: clinical pulmonary infection score;
ROC: Receiver operating characteristic curves.
